# Anti-Zika candidates from a marine fungus with a remarkable biosynthetic repertoire

**DOI:** 10.1016/j.jbc.2021.101047

**Published:** 2021-08-04

**Authors:** Ketan D. Patel, Andrew M. Gulick

**Affiliations:** Department of Structural Biology, Jacobs School of Medicine and Biological Sciences, University at Buffalo, Buffalo, New York, USA

**Keywords:** CDP, cyclodepsipeptide, DTX, destruxin, HIC, α-D-hydroxyisocaproic acid, ISD, isaridin, ISR, isariin, NRPS, nonribosomal peptide synthetase, 3MP, (3S)-methyl-L-proline

## Abstract

The study of natural products provides exciting opportunities for the discovery of novel biologically active molecules and biosynthetic pathways. Recently, Yuan and colleagues described 30 cyclic depsipeptides that are biosynthesized by proteins encoded by three distinct gene clusters in the marine fungus, *Beauveria felina*. Genetic and biochemical studies confirmed the involvement of nonribosomal peptide synthetases in the production of multiple compounds, some of which inhibit Zika virus replication.

Recent outbreaks caused by Zika virus, a member of the positive strand RNA flavivirus genus, have caused alarm notably due to the transmissibility of the virus across the placental barrier. Vaccines against Zika virus are still under development, and there are currently no approved antiviral treatments. Microbial natural products are an important source and inspiration for the development of novel pharmaceuticals, including as antiviral compounds (1). Many natural products originally identified during a period of in-depth investigation between the 1960s and 1980s are now being reexamined following advances in microbial culturing and genomic sequencing, as well as advances in biochemical and heterologous methods for production of these compounds. During the past decade, our understanding of the biosynthesis pathways that produce these compounds has increased, helping to realize the long-standing goal of predicting products from gene sequences and engineering or combining catalysts from different biosynthetic gene clusters to produce novel compounds with desirable activities (2).

Many peptide natural products are produced by the nonribosomal peptide synthetases (NRPSs), a family of megasynthetase proteins that produce peptides using an unusual enzymatic architecture. Generally, NRPSs consist of fused catalytic domains that function with an assembly line strategy to produce a variety of peptide natural products, including antibiotics, siderophores, signaling molecules, toxins, and antitumor molecules (3). Independent of ribosomes, mRNAs, and tRNAs, the NRPS enzymes can incorporate nonproteinogenic amino acids into their products. An important class of NRPS products is the cyclodepsipeptides (CDPs), which are cyclic arrangements of amino and hydroxy acids joined with amide and ester bonds (4). One family of cyclohexadepsipeptides includes destruxins (DTXs), isaridins (ISDs), and isariins (ISRs), which all contain six building blocks, including one β-amino or β-hydroxy acid, to form a 19-membered macrocycle. Multiple DTX, ISD, and ISR analogs exhibit diverse insecticidal, antifungal, antiviral, and antibacterial activities, and understanding the enzymatic basis of CDP biosynthesis can provide opportunities to develop new drugs with enhanced activities.

In a recent article in the *Journal of Biological Chemistry*, Yuan *et al.* (5) reported the discovery and characterization of biosynthetic gene clusters of several CDPs from the sea-sponge-associated fungus *Beauveria felina*. As part of an ongoing antiviral discovery campaign, a crude extract *Beauveria* was found to exhibit anti-Zika virus activity. The extract was subjected to NMR analysis as well as classification *via* the Global Natural Product Social (GNPS) molecular network resource (6), which serves as a public database for reference mass spectrometry data with crowd-sourced annotation. The clustering of observed spectra identified CDPs similar—and in some cases identical—to previously identified DTXs, ISDs, and ISRs. A total of 30 unique CDPs were identified and isolated, harboring nonproteinogenic amino acid building blocks, including β-alanine, (3*S*)-methyl-L-proline (3MP), and a variety of α-hydroxy acids such as α-D-hydroxyisocaproic acid (HIC) that form the single ester linkage of each CDP. Twenty-six of the compounds were shown to have low cytotoxicity against a lung epithelial cell line and were therefore tested in a Zika virus replication model. Seven active compounds had a significant inhibitory effect on viral replication as monitored by quantification of viral RNA and the production of an abundant viral nonstructural protein. A subset of these compounds was further examined and shown to be effective at an early stage of viral replication, potentially by blocking endosome acidification. Curiously, six of the seven active compounds contained modified HIC residues, suggesting this may be important for the observed antiviral activity.

The *B. felina* genome sequence reported by Yuan and colleagues identified 23 genes that encode NRPS proteins, three of which contain the six modules necessary for the production of CDPs containing six building blocks (Fig. 1). Bioinformatic analysis and gene deletion studies allowed the authors to assign all of the CDP products to the responsible biosynthetic gene clusters. Biosynthetic pathways were described to produce the different analogs from each cluster, and experimental evidence was provided to confirm the necessary auxiliary enzymes for producing 3MP and for modifying the HIC residues. 3MP has been observed in other nonribosomal and ribosomally encoded peptides, which respectively form the 3MP residue before or after peptide formation. In the production of the *Beauveria* CDPs, the Fe/α-ketoglutarate-dependent oxygenase DetxE was confirmed *via* gene deletion and biochemical assays to convert isoleucine to 4-methyl-Δ^1^-pyrroline-5-carboxylate. The final conversion to 3MP was again demonstrated with both genetic and biochemical experiments, which implicated one of two *Beauveria* Δ^1^-pyrroline-5-carboxylate reductases (P5CRs) in the formation of 3MP. *B. felina* therefore exploits the enzymes from primary metabolism to expand the diversity of CDPs that are produced. Understanding the rules that enable some organisms to incorporate enzymes from other pathways will expand our ability to predict products from genome sequences and engineer the production of novel compounds through heterologous or systems biology approaches.

**Figure 1:**
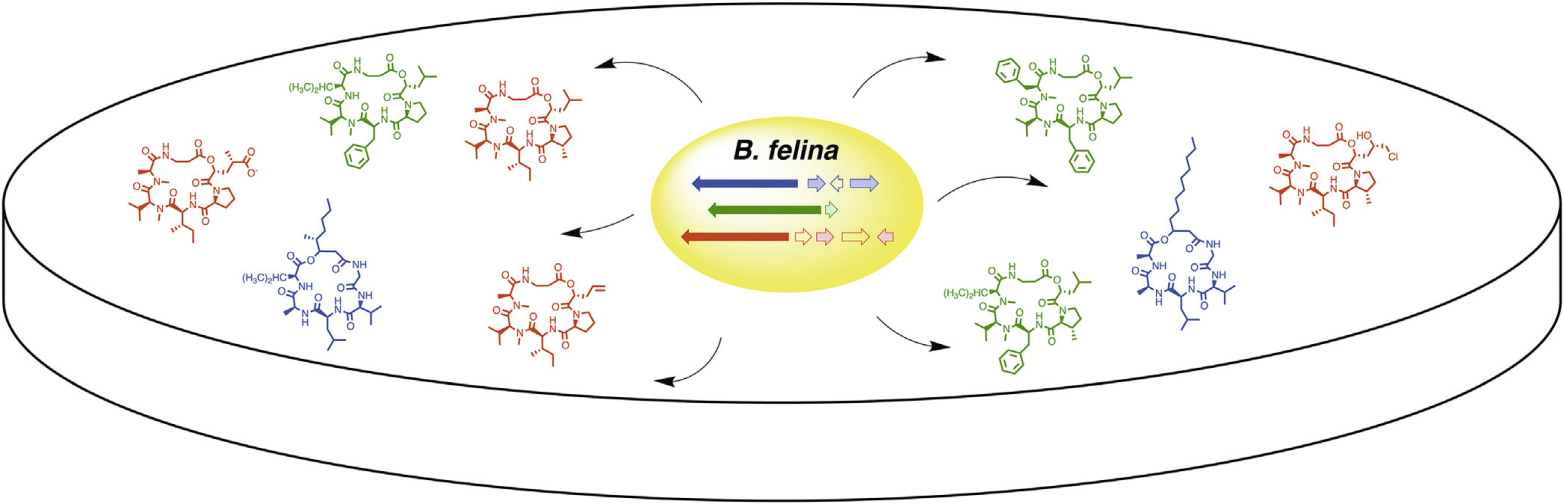
The marine sponge-associated fungus *B. felina* uses three biosynthetic pathways that involve large modular NRPS enzymes to produce as many as 30 cyclohexadepsipeptides, harboring nonproteinogenic amino acids and α-hydroxyacids. Several compounds showed promising anti-Zika virus activity.

The modular nature of the NRPS enzymes has raised the possibility that novel enzyme assembly lines could be designed by mutating or rearranging specific domains or modules. Recent structures of multidomain NRPS enzymes have fostered exciting progress in these efforts (7). In particular, the identification of tractable junction points for combinatorial engineering has enabled the formation of hybrid NRPSs from closely related systems to produce novel peptide products at high titres in several model systems (8, 9). The similarity of the fungal CDP systems provides an opportunity to diversify further the depsipeptide structures and produce additional compounds. Moreover, the reported biosynthesis of 3MP illustrates how a deeper understanding of the interactions of primary and secondary metabolic pathways in natural product biosynthesis can perhaps be used to incorporate necessary catalytic steps to expand the diversity of final products or improve the yield of important compounds. The mechanistic importance of the methyl group of the 3MP is as yet not known.

It is noteworthy that the majority of the compounds that prove most promising in the anti-Zika assays were destruxins that contain tailoring modifications that appear to be initiated by an oxidation by a Cytochrome P450 at the HIC residue (5). The mechanistic basis of this antiviral activity remains unknown; however, potential modifications to other inactive CDPs using either the *B. felina* P450 or other heterologous catalysts may identify additional compounds with desired activities. This recent study from Yuan *et al.* (10) expands the arsenal of biologically active natural products from the sponge-associated microbiome, which includes both ribosomally derived and nonribosomal peptides. These studies should inspire future screening of natural products, microbial culturing, and genome mining of unique microbes such as sponge-associated fungi or uncultured bacteria from diverse environments to discover novel natural products or new derivatives of known compounds with greater efficacy.

## Conflicts of interest

The authors declare that they have no conflicts of interest with the contents of this article.
